# Promoting Safe Sleep, Tobacco Cessation, and Breastfeeding to Rural Women During the COVID-19 Pandemic: Quasi-Experimental Study

**DOI:** 10.2196/31908

**Published:** 2021-11-22

**Authors:** Carolyn R Ahlers-Schmidt, Christy Schunn, Ashley M Hervey, Maria Torres, Jill Elizabeth V Nelson

**Affiliations:** 1 Center for Research for Infant Birth and Survival University of Kansas School of Medicine-Wichita Wichita, KS United States; 2 Department of Pediatrics University of Kansas School of Medicine-Wichita Wichita, KS United States; 3 Kansas Infant Death and SIDS Network Wichita, KS United States; 4 Delivering Change Inc Junction City, KS United States

**Keywords:** COVID-19, SIDS, sudden infant death syndrome, safe sleep, tobacco cessation, breastfeeding, virtual education

## Abstract

**Background:**

Safe Sleep Community Baby Showers address strategies to prevent sleep-related infant deaths. Due to the COVID-19 pandemic, these events transitioned from in-person to virtual.

**Objective:**

This study describes outcomes of transitioning Safe Sleep Community Baby Showers to a virtual format and compares outcomes to previous in-person events.

**Methods:**

Participants from four rural Kansas counties were emailed the presurvey, provided educational materials (videos, livestream, or digital documents), and completed a postsurvey. Those who completed both surveys received a portable crib and wearable blanket. Within-group comparisons were assessed between pre- and postsurveys; between-group comparisons (virtual vs in-person) were assessed by postsurveys.

**Results:**

Based on data from 145 in-person and 74 virtual participants, virtual participants were more likely to be married (*P*<.001) and have private insurance (*P*<.001), and were less likely to report tobacco use (*P*<.001). Both event formats significantly increased knowledge and intentions regarding safe sleep and avoidance of secondhand smoke (all *P*≤.001). Breastfeeding intentions did not change. Differences were observed between in-person and virtual meetings regarding confidence in the ability to avoid secondhand smoke (in-person: 121/144, 84% vs virtual: 53/74, 72%; *P*=.03), intention to breastfeed ≥6 months (in-person: 79/128, 62% vs virtual: 52/66, 79%; *P*=.008), and confidence in the ability to breastfeed ≥6 months (in-person: 58/123, 47% vs virtual: 44/69, 64%; *P*=.02).

**Conclusions:**

Although both event formats demonstrated increased knowledge/intentions to follow safe sleep recommendations, virtual events may further marginalize groups who are at high risk for poor birth outcomes. Strategies to increase technology access, recruit priority populations, and ensure disparities are not exacerbated will be critical for the implementation of future virtual events.

## Introduction

The impact of SARS-CoV-2 on maternal and perinatal outcomes appears to be less severe than initially thought, though infection is still a cause for concern [1-4]. However, impacts appear to go beyond the physiologic reactions to direct infection [1]. Pregnant and postpartum women have reported changes in employment and financial status, mental health, social support, and for some even access to care [5]. Women also reported changes in infant care practices, such as breastfeeding and infant sleep strategies, specifically attributed to the pandemic, though changes did not always reach statistical significance [5].

Although empirical data are not yet available, personal communication with emergency and support services indicate there may be an increase of sleep-related infant deaths during the pandemic. Sleep-related infant deaths, including sudden infant death syndrome (SIDS), accidental suffocation or strangulation in bed, and other undetermined deaths, are the primary cause of death for infants from 28 days to 1 year of life despite risk reduction strategies promoted by the American Academy of Pediatrics (AAP; eg, supine position) [6]. Programs such as Safe Sleep Community Baby Showers [7-9] are a recognized strategy to promote infant safe sleep [10] where women and their support persons are brought together at a community venue to celebrate their pregnancy and receive education. Topics address risk reduction strategies to prevent sleep-related infant deaths, including safe sleep position and surface, breastfeeding, and tobacco-free environments. Tools needed to create a safe sleep environment (eg, portable crib or wearable blanket) are often provided to attendees [7-9].

During the COVID-19 pandemic, many programs that support maternal and infant health, including education on the AAP safe sleep recommendations, had to redirect resources and reduce or even halt support services. New delivery strategies were needed to accommodate stay-at-home orders and gathering size restrictions when services were available. One such strategy was virtual education; however, the impact of transitioning Safe Sleep Community Baby Showers from in-person to virtual is unknown. As such, the purpose of this study is to describe the outcomes of virtual Safe Sleep Community Baby Showers and compare the results to previous in-person events.

## Methods

### Settings

The Kansas Infant Death and SIDS (KIDS) Network has created a statewide infrastructure of certified safe sleep instructors [8,11] who facilitate in-person Safe Sleep Community Baby Showers. With the support of the KIDS Network, safe sleep instructors in four rural counties (Geary, Cloud, Harvey, and Shawnee) held virtual Safe Sleep Community Baby Showers in 2020. Outcomes from these events were compared to previous in-person Safe Sleep Community Baby Showers held in 2019.

### Participants

Participants were pregnant or postpartum women. For in-person events, participants were recruited via social media, radio ads, and fliers, and through health care providers and maternal and child health programs. Presurveys were completed on paper at the event prior to the education. Postsurveys were completed immediately following the education. Participants for virtual events were recruited through local outreach including social media and referral by partner programs and events. Potential participants were emailed a link and instructions to complete the presurvey. Once completed, educational materials and links were distributed. The postsurvey link with instructions was emailed following completion of the education. Participants at all events who completed both pre- and postsurveys received a portable crib and wearable blanket.

### Instruments

A 22-item presurvey, including demographics; knowledge; intention; and practice questions on safe sleep, tobacco use/avoidance, and breastfeeding, was completed by participants prior to receiving education. Due to skip logic, not all participants completed all items. At the end of the event, 13 of the same knowledge and intention items from the presurvey and an additional 9 items related to confidence and satisfaction with the event were collected. Deidentified survey data were collected and managed using REDCap, a secure web-based data capture application hosted at the University of Kansas Medical Center [12,13].

### Education

Safe sleep, breastfeeding, and tobacco cessation/avoidance education was provided to participants regardless of education format. In-person events were interactive by nature, using presentation and demonstration, but also included video components. For virtual events, Geary and Cloud counties chose to provide educational videos and prerecorded presentations to participants (passive). Harvey and Shawnee counties held real-time interactive education over a virtual platform (interactive).

### Statistical Analysis

Descriptive statistics, confidence items, and satisfaction are summarized using frequencies (percentages). Comparisons between pre- and postsurveys were made using McNemar test for paired dichotomous variables (safe vs unsafe responses), Friedman test, and chi-square likelihood ratio test. Data from previous in-person Safe Sleep Community Baby Showers for three of the four counties were used to assess potential differences in postintervention outcomes. One was omitted due to using a previous version of the survey. The Mann-Whitney Wilcoxon test for independent samples was used for comparison between virtual and in-person events. Due to different education formats (interactive and passive) for virtual Safe Sleep Community Baby Showers, a secondary data analysis was completed. Alpha was set a priori at .05. Statistical analyses were performed using SPSS for Windows, Version 23.0 (IBM Corp). This project involved secondary analysis of deidentified program data and was reviewed by the University of Kansas Medical Center Human Subjects Committee who determined it to not be human participant research.

## Results

### Participants

Between August 2020 and November 2020, four virtual Safe Sleep Community Baby Showers were held in rural Kansas counties: Harvey, Geary, Cloud, and Shawnee. A total of 97 individuals engaged in the virtual events; 22 completed only the presurvey, and 1 completed only the postsurvey. Therefore, 74 participants were included in the analysis. Due to similarity in results between events, data is reported in aggregate on the tables. In 2019, one in-person Safe Sleep Community Baby Shower was held in each of the following counties Geary, Cloud, and Shawnee counties with a total of 145 attendees across all events. All completed both pre- and postsurveys.

### Demographics

Full demographics are in Table 1. Differences in marital status and insurance status were observed between virtual and in-person participants. Virtual participants were significantly more likely to be married (*P*<.001) and have private insurance (*P*<.001).

**Table 1:** Participant characteristics.^a^

		In-person CBS^b^ (n=145), n (%)	Virtual CBS (n=74), n (%)	Between group difference, *P* value^c^	
**County of residence**					<.001
	Harvey	0 (0.0)	15 (20.3)		
	Geary	54 (37.2)	42 (56.8)		
	Cloud	20 (13.8)	11 (14.9)		
	Shawnee	71 (49.0)	6 (8.1)		
**Race/ethnicity**					.44
	Non-Hispanic White	87 (60.4)	51 (68.9)		
	Non-Hispanic Black	30 (20.8)	10 (13.5)		
	Hispanic	15 (10.4)	9 (12.2)		
	Other^d^	12 (8.3)	4 (5.4)		
**Marital status**					<.001
	Single	58 (40.3)	8 (10.8)		
	Married	59 (41.0)	51 (68.9)		
	Other^e^	27 (18.8)	15 (20.3)		
**Partner race/ethnicity**					.64
	Non-Hispanic White	74 (51.0)	46 (62.2)		
	Non-Hispanic Black	27 (18.6)	11 (14.9)		
	Hispanic	17 (11.7)	7 (9.5)		
	Other^d^	14 (9.7)	5 (6.8)		
	Not applicable/choose not to answer	13 (9.0)	5 (6.8)		
**Mother’s education**					.05
	Some high school	23 (16.0)	5 (6.8)		
	High school graduate or GED^f^	79 (54.9)	32 (43.2)		
	2-year community college graduate	12 (8.3)	13 (17.6)		
	4-year college graduate	15 (10.4)	13 (17.6)		
	Graduate school	9 (6.3)	7 (9.5)		
	Other	6 (4.2)	4 (5.4)		
**Insurance status**					.001
	Private insurance	27 (18.8)	26 (35.1)		
	KanCare/Medicaid	84 (58.3)	23 (31.1)		
	Military	24 (16.7)	20 (27.0)		
	Other^g^	9 (6.3)	5 (6.8)		
**Prenatal care provider**					.11
	Private provider’s office	54 (37.8)	34 (46.6)		
	Hospital clinic	66 (46.2)	30 (40.5)		
	Community health clinic	16 (11.2)	4 (5.4)		
	Clinic at work or school	0 (0.0)	2 (2.7)		
	County health department	2 (1.4)	0 (0.0)		
	Other	5 (3.5)	3 (4.1)		

^a^Missing data: in-person: race/ethnicity (n=1), marital status (n=1), mother’s education (n=1), insurance status (n=1), prenatal care provider (n=2); virtual: prenatal care provider (n=1).

^b^CBS: Community Baby Showers.

^c^*P* value <.05 indicates a statistically significant difference between pre- and postsurvey responses.

^d^Race/ethnicity: other includes multiracial and other.

^e^Marital status: other includes partnered, separated, and divorced.

^f^GED: General Educational Development.

^g^Insurance status: other includes self-pay, managed care organization/marketplace, and other.

### Changes in Safe Sleep Knowledge and Intentions

Following the Safe Sleep Community Baby Showers, in-person participants demonstrated a positive increase from pre- to postsurvey in intention to follow safe sleep practices related to anticipated sleep position (pre: 128/144, 89% vs post: 142/144, 99%; *P*<.001), anticipated sleep surfaces (pre: 126/145, 87% vs post: 140/145, 97%; *P*=.001), anticipated crib items (pre: 86/130, 66% vs post: 123/130, 95%; *P*<.001), and discussing safe sleep with others (pre: 90/138, 65% vs post: 132/138, 96%; *P*<.001; Table 2). On the postsurvey, the majority (123/125, 98%) reported knowing at least one person who would support safe sleep. Virtual participants also demonstrated a positive increase from pre- to postsurvey in intention to follow safe sleep practices related to only placing their baby on the back to sleep (pre: 63/74, 85% vs post: 74/74, 100%; *P*=.001), safe sleep surfaces (pre: 60/73, 82% vs post: 71/73, 97%; *P*=.001), inclusion of only safe items in the crib (pre: 58/73, 80% vs post: 71/73, 97%; *P*<.001), and discussing safe sleep with others (pre: 53/73, 73% vs post: 73/73 100%; *P*<.001). In addition, all virtual participants (74/74, 100%) reported knowing at least one person who would support safe sleep. No differences in anticipated safe sleep practices were observed between those who attended an in-person event compared to those who attended a virtual event.

**Table 2:** Changes in intended safe sleep practices.^a^

	In-person CBS^b^ (n=145)			Virtual CBS (n=74)			Between-group differences, *P* value^c^
	Presurvey, n (%)	Postsurvey, n (%)	Within-group difference, *P* value	Presurvey, n (%)	Postsurvey, n (%)	Within-group difference, *P* value	
Safe sleep position (back only)	128 (88.9)	142 (98.6)	<.001	63 (85.1)	74 (100)	.001	.31
Safe sleep surface (crib, portable crib, or bassinet only)	126 (86.9)	140 (96.6)	.001	60 (82.2)	71 (97.3)	.001	.78
Safe crib items (firm mattress, fitted sheet, or wearable blanket only)	86 (66.2)	123 (94.6)	<.001	58 (79.5)	71 (97.3)	<.001	.33
Have or plan to discuss safe sleep with others	90 (65.2)	132 (95.7)	<.001	53 (72.6)	73 (100)	<.001	.07

^a^Missing data: in-person: sleep position (n=1), crib items (n=15), talk to others about safe sleep (n=7); virtual: sleep surface (n=1), crib items (n=1), talk to others about safe sleep (n=1).

^b^CBS: Community Baby Showers.

^c^*P* value <.05 indicates statistically significant difference between pre- and postsurvey responses.

### Changes in Readiness to Quit and Knowledge of a Tobacco-Free Environment

The majority of in-person participants (n=100, 69%) and virtual participants (n=72, 97%) reported not using tobacco products in the 6 months prior to the Safe Sleep Community Baby Showers; however, this number was significantly lower for in-person participants (*P*<.001). Of in-person participants reporting tobacco use (n=44/144), the majority (n=27/44, 61%) reported daily use, while 5% (n=2/44) reported weekly and 34% (n=15/44) were not currently using. Of virtual participants who reported using (n=2/74), one was not currently using and the other reported daily use. No significant changes in readiness to quit were observed between pre- and postsurvey for either group.

Positive changes were observed for in-person participants from pre- to postsurvey regarding plans to not allow tobacco use in the home or car (pre: 123/142, 87% vs post: 132/142, 93%; *P*=.04), knowledge of three ways to avoid secondhand exposure (pre: 107/140, 76% vs post: 135/140, 96%; *P*<.001), and knowledge of at least three local resources for tobacco cessation (pre: 24/133, 18% vs post: 55/133, 41%; *P*<.001; Table 3). Following the events, virtual participants also reported positive changes from pre- to postsurvey in plans to not allow tobacco use inside their home or car (pre: 67/74, 91% vs post: 73/74, 99%; *P*=.01), knowledge of three ways to avoid secondhand exposure (pre: 52/74, 70% vs post: 74/74, 100%; *P*<.001), and knowledge of at least three local resources for tobacco cessation (pre: 7/73, 10% vs post: 38/73, 52%; *P*<.001). No differences were observed between virtual and in-person participants.

**Table 3:** Smoking exposure, cessation, resources, and intent to quit.^a^

		In-person CBS^b^ (n=145)					Virtual CBS (n=74)						Between-group differences, *P* value^c^
		Presurvey, n (%)	Postsurvey, n (%)	Within-group difference, *P* value		Presurvey, n (%)		Postsurvey, n (%)	Within-group difference, *P* value				
**Secondhand exposure in home or car^d^**					.04					.01		.05	
	Never	123 (86.6)	132 (93.0)			67 (90.5)		73 (98.6)					
	Daily	18 (12.7)	9 (6.3)			5 (6.8)		1 (1.4)					
	Weekly	1 (0.7)	1 (0.7)			2 (2.7)		0 (0.0)					
**Know** ≥**3 ways to avoid secondhand exposure**					<.001					<.001		.10	
	Yes	107 (76.4)	135 (96.4)			52 (70.3)		74 (100)					
	No	33 (23.6)	5 (3.6)			22 (29.7)		0 (0.0)					
**Know** ≥**3 local resources for tobacco cessation**					<.001					<.001		.12	
	Yes	24 (18.0)	55 (41.4)			7 (9.6)		38 (52.1)					
	No	109 (82.0)	78 (58.6)			66 (90.4)		35 (47.9)					

^a^Missing data: in-person: secondhand exposure in home or car (n=3), know >3 ways to avoid secondhand exposure (n=5), know >3 local resources (n=12); virtual: know >3 local resources (n=1).

^b^CBS: Community Baby Showers.

^c^*P* value <.05 indicates statistically significant difference between pre- and postsurvey responses.

^d^Presurvey indicates actual behavior; postsurvey represents future intention.

### Changes in Breastfeeding Intentions

In-person participants planned to breastfeed their baby with no change observed from pre- to postsurvey (pre: 130/138, 94% vs post: 132/138, 96%; *P*=.53; Table 4). Differences were also not observed in intention to breastfeed longer than 6 months (pre: 77/128, 60% vs post: 79/128, 62%; *P*=.63). However, following the events, more in-person participants reported being confident in their ability to breastfeed for longer than 6 months (pre: 50/123, 41% vs post: 58/123, 47%; *P*=.008), and knowledge of at least three local breastfeeding resources (pre: 45/138, 33% vs post: 81/138, 59%; *P*<.001). Virtual participants planned to breastfeed their baby with no change observed pre- to postsurvey (pre: 69/74, 93% vs post: 69/74, 93%; *P*=.56). No differences were reported in intention to breastfeed longer than 6 months (pre: 52/66, 79% vs post: 52/66, 79%; *P*>.99) or confidence in ability to breastfeed longer than 6 months (pre: 41/69, 59% vs post: 44/69, 64%; *P*=.38). A statistically significant difference was observed in knowledge of at least three local breastfeeding resources (pre: 13/74, 18% vs post: 41/74, 55%; *P*<.001) following the virtual events. Differences were observed between in-person and virtual participants in their intention to breastfeed longer than 6 months (post: 79/128, 62% vs post: 58/66, 79%; *P*=.008) and confidence in ability to breastfeed for longer than 6 months (post: 58/123, 47% vs post: 44/69, 64%; *P*=.02).

**Table 4:** Breastfeeding intent, confidence, and knowledge of resources.^a^

			In-person CBS^b^ (n=145)							Virtual CBS (n=74)									Between-group differences, *P* value^c^	
			Presurvey, n (%)		Postsurvey, n (%)		Within-group difference, *P* value			Presurvey, n (%)			Postsurvey, n (%)			Within-group difference, *P* value				
**Likelihood of breastfeeding**								.53									.56			.80
	Don’t plan to breastfeed	4 (2.9)		5 (3.6)					5 (6.8)			5 (6.8)								
	Not likely	4 (2.9)		1 (0.07)					0 (0.0)			0 (0.0)								
	Somewhat likely	25 (18.1)		24 (17.4)					10 (13.5)			11 (14.9)								
	Very likely	105 (76.1)		108 (78.3)					59 (79.7)			58 (78.4)								
**Intend to breastfeed >6 months**								.63									>.99			.008
	Yes	77 (60.2)		79 (61.7)					52 (78.8)			52 (78.8)								
	No	51 (39.8)		49 (38.3)					14 (21.2)			14 (21.2)								
**Confident in ability to breastfeed for >6 months**								.008									.38			.02
	Yes	50 (40.7)		58 (47.2)					41 (59.4)			44 (63.8)								
	No	73 (59.3)		65 (52.8)					28 (40.6)			25 (36.2)								
**Knowledge of** ≥**3 local breastfeeding resources**								<.001									<.001			.65
	Yes	45 (32.6)		81 (58.7)					13 (17.6)			41 (55.4)								
	No	93 (67.4)		57 (41.3)					61 (82.4)			33 (44.6)								

^a^Missing data: in-person: likelihood (n=7), duration (n=6), confidence (n=11), knowledge of local resources (n=7); virtual: duration (n=8), confidence (n=5).

^b^CBS: Community Baby Showers.

^c^*P* value <.05 indicates statistically significant difference between pre- and postsurvey responses.

### Confidence Change

On the postsurvey, participants were asked to rate their confidence based on education received (Table 5). Significant differences were only observed between the two groups in confidence to avoid secondhand smoke (*P*=.03).

**Table 5:** Confidence in ability to engage in risk reduction strategies following Safe Sleep Community Baby Showers.^a^

		In-person CBS^b^ (n=145)	Virtual CBS (n=74)	Between-group difference, *P* value^c^
**Get baby to sleep on their back**				.22
	Less confident	1 (0.7)	0 (0.0)	
	No change	24 (16.6)	18 (24.3)	
	More confident	120 (82.8)	56 (75.7)	
**Have baby sleep in my room, but separate crib, portable crib, or bassinet**				.18
	Less confident	1 (0.7)	0 (0.0)	
	No change	23 (15.9)	18 (24.3)	
	More confident	121 (83.4)	56 (75.7)	
**Keep loose blankets out of the crib**				.60
	Less confident	3 (2.1)	0 (0.0)	
	No change	25 (17.4)	17 (23.0)	
	More confident	116 (80.6)	57 (77.0)	
**Follow safe sleep recommendations even when people give different advice**				.50
	No change	17 (15.2)	14 (18.9)	
	More confident	95 (84.8)	60 (81.1)	
**Avoid secondhand smoke**				.03
	No change	23 (16.0)	21 (28.4)	
	More confident	121 (84.0)	53 (71.6)	
**Breastfeed**				.14
	No change	36 (25.5)	26 (35.1)	
	More confident	105 (74.5)	48 (64.9)	

^a^Missing data: in-person: loose blankets (n=1), follow recommendations (n=33), secondhand smoke (n=1), breastfeeding (n=4).

^b^CBS: Community Baby Showers.

^c^*P* value <.05 indicates statistically significant difference between pre- and postsurvey responses.

### Participant Satisfaction

Satisfaction with events was high. In-person participants were very satisfied (120/144, 83%), satisfied (22/144, 15%), or neutral (2/144, 1%). The majority of virtual participants reported being very satisfied (57/74, 77%). The remainder were satisfied (16/74, 22%) or neutral (1/74, 1%). Several comments specifically addressed the virtual nature of the training. One woman stated:

Thank you for the opportunity to participate in the community baby shower over zoom! It's a great way to keep promoting safe sleep for babies while keeping up with the strange times we are living in today.

No significant differences in event satisfaction were observed between in-person and virtual participants (*P*=.27).

### Secondary Analysis of Virtual Education Formats

Two different education formats were used at the virtual Safe Sleep Community Baby Showers. A total of 53 (71.6%) participants received passive education and 21 (28.4%) attended an interactive virtual event. Participants who attended passive virtual events were significantly more likely to have received a high school diploma or General Educational Development (GED; *P*=.01) and have military insurance (*P*=.01), whereas participants who attended interactive events were more likely to receive prenatal care at a private provider’s office (*P*=.01). No differences in anticipated safe sleep practices, smoking exposure or cessation, breastfeeding intention or confidence, or confidence on engagement in risk reduction strategies were observed between those who attended a passive virtual event compared to those who attended an interactive virtual event. Differences between the two groups were observed regarding knowledge of resources following the events. Specifically, participants who attended interactive events were more likely to know three or more local resources for tobacco cessation (*P*<.001) and three or more local breastfeeding resources (*P*<.001).

## Discussion

### Impact of Virtual Format

Safe Sleep Community Baby Showers held as virtual events in rural counties due to the COVID-19 pandemic had significantly more participants who reported being married and on private insurance than in-person events. These characteristics are frequently associated with positive perinatal outcomes (eg, [14,15]). In addition, though it did not cross the threshold for significance, virtual attendees were less likely to report low education levels (37/74, 50% high school diploma/GED or less) than in-person attendees (102/144, 71%).

Women of higher socioeconomic status may have been more likely to participate in Safe Sleep Community Baby Showers for a variety of reasons. Rural communities are highly susceptible to COVID-19 due to vulnerable populations, fewer physicians, and lack of related services [16]. However, impacts may be especially dire for socially vulnerable populations [16], and concerns for immediate needs (eg, food, housing, or employment) impacted by the pandemic may have resulted in lower participation in educational events by low-income women. Further, during the pandemic, many health departments and health care providers had to modify or suspend services such as prenatal home visits, which may have promoted Safe Sleep Community Baby Showers to hard-to-reach families.

Differences in participants between the two event formats may also highlight access disparities that are exacerbated with the use of technology [17]. To reduce unintended negative impacts, future events could use Crawford and Serhal’s [18] digital health equity framework, an expansion of Dover and Belon’s [19] theories of health equity. Dover and Belon’s [19] model suggests that the interplay of social determinants of health and health system use impact health equity. Within the model, impacts of socioeconomic, cultural, and political context, and their influence on the social stratification process, health policy context, environment, health-related behaviors and health beliefs, and social circumstances are explored [19]. Crawford and Serhal [18] expand this framework by considering the impacts of digital health resources and digital health literacy in enhancing health equity. For example, an individual’s use of technology and capacity to access and interpret digital content is shaped by their social, cultural, and economic position, which should be considered in the development of health care and education and, even more importantly, in the development of policy [18].

As COVID-19 transmission risks are reduced through increased vaccine availability, it may be important to consider ways to safely hold in-person events, as data suggests these events serve individuals reporting more sociodemographic and behavioral risk factors associated with infant mortality [20]. If COVID-19 risks persist, identifying outreach strategies and partnerships to increase access to technology may be critical to ensure high-risk families have access to virtual events and to prevent further marginalizing disparate groups. Event dissemination and recruitment strategies may also need to be shifted to better promote virtual events to disadvantaged groups, such as through health care providers, other maternal child health programs, or trusted community members.

Despite demographic differences in attendees, both event formats were successful at promoting the AAP Safe Sleep Recommendations, with participants showing significant increases regarding intentions to use safe sleep practices following the baby showers. Postevent rates reflected those from previously published studies [7-9]. Similarly, positive improvements were observed within events for tobacco cessation/avoidance items, though self-reported tobacco use was significantly higher for in-person participants. This could further reflect in-person participation by a higher risk group or may suggest a higher likelihood to truthfully report tobacco use in person. Fewer improvements were observed for breastfeeding intention and duration, though knowledge of breastfeeding support resources increased. In addition, only the in-person events increased participant confidence in the ability to breastfeed for greater than 6 months, which has been linked to benefits for both mother and infant, including reduced infant mortality [21].

To further assess impacts of the virtual education, a secondary analysis was performed to compare passive versus active education strategies. Participants differed in terms of demographic variables such as insurance type, but this is likely a reflection of the community at large and not the educational format. For example, Geary County, which used a passive education format, had high rates of military insurance but is the home of a military base. In terms of knowledge outcomes, the most prominent difference appeared in recognition of tobacco cessation and breastfeeding support resources. This may have resulted from additional discussion by participants and presenters in the interactive format. If the passive format will be used in the future, special care should be taken to provide additional information on resources available to support desired behaviors.

### Limitations

This study is limited as events took place in rural counties in a Midwest state and may not be generalizable to urban areas or other regions. These rural communities had been engaged in safe sleep promotion through the Safe Sleep Instructor [8,11] project over a number of years, which may have impacted baseline data and openness to safe sleep education. The proportions of participants by county differed between in-person and virtual formats, which may have contributed to demographic differences. However, poverty rates for the counties were comparable: Harvey 9.6%, Cloud 11.4%, Shawnee 11.4%, and Geary 13%; state range 3.3% to 22.4% [22]. Data were self-reported, which could result in social desirability response bias. In addition, behavioral data following the event could not be collected, as it was outside the scope of this project. Future studies should assess parent behaviors related to infant safe sleep following educational events. The authors would like to note there were fewer missing data with the virtual trainings. This may indicate a benefit of allowing participants to complete data forms at their leisure prior to the event. Future research should assess attitudes and comfort around completing surveys online compared to in-person.

### Conclusions

Although both event formats demonstrated the ability to increase knowledge/intentions in most areas measured, virtual events may further marginalize groups who are at high risk for poor birth outcomes. These findings have implications beyond safe sleep promotion, especially as the COVID-19 pandemic continues to accelerate the use of telemedicine and virtual platforms for public health education. Strategies to increase technology access, recruit priority populations, and ensure disparities are not enhanced will be critical for implementation of future virtual events.

## Abbreviations

AAP: American Academy of Pediatrics
GED: General Educational Development
KIDS: Kansas Infant Death and SIDS
SIDS: sudden infant death syndrome
